# A Case of Ossification of the Placenta, Occurring Several Times in the Same Individual

**Published:** 1852-03

**Authors:** Charles Garrison

**Affiliations:** Swedesboro’, New Jersey


*A Case of Ossification of the Placenta, occurriug several times in the same Individual.* By Charles Garrison, M. D., Swedesboro', New Jersey.—Most of our writers on obstetrics have treated at some length on the subject of retention of the placenta, its causes and effects; but I have not been able to find in any of them a satisfactory account of that form of retention which is caused by ossification of the uterine surface of the placenta. It is, however, eminently deserving our attention, both from the interest which attaches to the questions relating to the reasons and mode of its production, and from the formidable nature of the difficulties and dangers to which it may give rise in actual practice. These latter are well illustrated in the following case, which, although constituting my entire experience on this point, has, nevertheless, been so peculiar in some of its features, that it may serve to add a little to our yet small stock of information upon this subject.

On the 25th of October, 1835, I was called to attend Mrs. J. H., in her second confinement. She stated to me that, in her first labor, which had occurred some three years previously, there had been great difficulty, particularly with the placenta; that the physician in attendance had been obliged to send to Philadelphia for Dr. Meigs, who ultimately delivered it, though with great pain to her and apparent difficulty to himself, and not till after the occurrence of considerable hemorrhage. What had been the nature of the obstacle she did not know.

On examination, I found the presentation favorable, and the labor natural; the child was rapidly pushed down, and in about four hours the delivery was accomplished. After the birth of the child, I applied my hand to the abdomen, and felt the uterus strongly contracted into a ball of the ordinary size, and began to think that, whatever untoward accident might have complicated the former labor, there was not likely to be any trouble with this. But in about ten minutes there was a tremendous gush of blood, which was quickly followed by another quite as large as the first, the result of which was great and almost fatal prostration; the pulsations at the wrist were scarcely felt, the face became pale, the hands cold, and the respiration sighing; she grew sick and vomited, complained of being very cold, pushed down the bed clothes, gasped for breath, and in a very short time after the last discharge was in a profound syncope. In the mean time, I had poured a pitcher of iced water, which was previously prepared, over the abdomen, and introduced my hand into the uterus, quickly and without any obstacle, as the hemorrhage had produced complete relaxation of both the uterus and external parts; but on reaching the placenta, and attempting to introduce my fingers between it and the uterus, for the purpose of effecting its detachment, I found it impossible to do so, as the union between them seemed perfectly firm and unyielding. After a moment's consideration, I determined to make a separation at all hazards, if it was possible, as death was inevitable if the placenta should be long retained. I began the operation slowly and cautiously, as the separation could only be accomplished by actually tearing or scratching the placenta from the surface of the womb, which could not be done except by the exertion of considerable force, more than it seemed possible that she could survive; and it was accompanied by a kind of crackling noise, which was heard distinctly both by myself and the attendants at the bedside. I soon found that I must hasten the delivery, or the patient would die before it was effected, and, letting go the cord, I placed my hand over the outside of the uterus, and, as rapidly as I was able, proceeded to loosen the placenta from its attachments. In this manner I succeeded in separating the whole mass, though in detached portions, which, together with my hand, were soon expelled by a smart contraction of the uterus. But although I had succeeded in removing the placenta; I still felt extremely anxious about the patient; she was yet in a state of complete unconsciousness. I gave her a *large* dose of opium, applied ice to the abdomen, and kept up frictions over the outside of the uterus till it was firmly contracted and all hemorrhage had ceased. She roused up for a moment, but the syncope immediately returned, and it was a considerable length of time before she showed any persistent signs of reviving animation.

On examining the placenta, I found all that surface of it which came in contact with the uterus in a state of ossification; it presented, through a common pocket glass, a kind of cellular arrangement, similar in appearance to what might be produced by sticking the whole surface full of the husks of wheat which had been broken in two, leaving the broken edge of the husk turned outward. It was undoubtedly the separation of this bony matter which gave rise to the crackling noise which had been heard during the process of delivery. I could reproduce the same sound in the placenta after it was expelled; it crackled in my fingers like frozen grass or ground. I had never upon any previous occasion been compelled to use an equal degree of force in the delivery of the placenta, and I felt very doubtful as to what might be the issue of it; the utmost strength of my fingers was barely able to break up this bony union.

Since this time I have attended Mrs. H. in *five confinements*, and in all but one there was a similar condition of the afrer-birth, accompanied by similar phenomena of excessive flooding and great difficulty of separating the bony surfaces of the uterus and placenta. In that one, her fourth labor, I did not reach her till the child had been delivered by a midwife, who, in her zeal to complete the labor before my arrival, by removing the after birth, had pulled so hard upon the cord that she had torn it quite away, leaving the placenta still within the womb; but in this instance there was *not any hemorrhage*, which led me to hope that it was merely a case of ordinary retention, without any formation of ossific matter, and on the introduction of my hand, I was pleased to find that my conjecture had been right. The placenta was easily detached and soon expelled; had there been the same difficulties here as in all the other labors through which I attended her, she must, in all probability, have lost her life before she could have been delivered.

Her last labor occurred about two years since, accompanied with the same circumstances as had characterized the other instances in which bony adhesions had taken place. I did not reach her until after the birth of the child, and, although the placenta was delivered as quickly as was possible in so difficult a case, yet the amount of hemorrhage had been such before my arrival, that for many hours I altogether despaired of her recovery from the terrible syncope which it had induced; but, under the use of laıge and repeated doses of opium, she at length revived, and soon regained her usual amount of strength. —*Amer. Jour. Med. Science.*

